# Parent-assessed quality of life among adolescents undergoing orthodontic treatment: a 12-month follow-up

**DOI:** 10.1590/2177-6709.20.5.094-100.oar

**Published:** 2015

**Authors:** Lucas Guimarães Abreu, Camilo Aquino Melgaço, Mauro Henrique Nogueira Guimaraes Abreu, Elizabeth Maria Bastos Lages, Saul Martins Paiva

**Affiliations:** 1 PhD student, Universidade Federal de Minas Gerais (UFMG), Department of Pediatric Dentistry and Orthodontics, Belo Horizonte, Minas Gerais, Brazil; 2 Professor of Orthodontics, UNINCOR, Belo Horizonte, Minas Gerais, Brazil; 3 Professor, Universidade Federal de Minas Gerais (UFMG), Department of Community and Preventive Dentistry, Belo Horizonte, Minas Gerais, Brazil; 4 Professor, Universidade Federal de Minas Gerais (UFMG), Department of Pediatric Dentistry and Orthodontics, Belo Horizonte, Minas Gerais, Brazil

**Keywords:** Parents, Caregivers, Adolescent, Quality of life, Orthodontic appliances

## Abstract

**Objective:**

To assess parents' and caregivers' view of the first twelve months of adolescents' orthodontic treatment with fixed appliances and to assess the evaluative properties of the Brazilian version of the Parental-Caregiver Perceptions Questionnaire (P-CPQ) in the orthodontic setting.

**Methods:**

Data from a sample of 96 parents and caregivers of adolescents undergoing orthodontic treatment with fixed appliances were collected by means of P-CPQ. Assessments were performed before banding and bracket bonding (T_1_) and 12 months after placement of fixed appliances (T_2_). Statistical analysis included Wilcoxon signed-rank test for the overall P-CPQ score and Bonferroni correction for P-CPQ subscales. The evaluative properties of the P-CPQ were assessed through responsiveness calculation and the minimally clinical important difference (MCID).

**Results:**

Among the 96 participants, 76 were mothers of patients, 16 were fathers, and four were other family members. Adolescents' mean age was 11.49 ± 0.50 years. Most families earned equal to or less than three times the Brazilian monthly minimum wage. There was significant improvement in the emotional and social well-being subscales (*p *< 0.001), which contributed to improve patient's overall quality of life (*p*< 0.001). Reductions in scores were associated with clinically meaningful moderate changes in the overall score as well as in the emotional and social well-being subscales. The MCID was 6.16 for the P-CPQ overall score.

**Conclusion:**

Parents and caregivers reported significant improvement in the quality of life of adolescents undergoing orthodontic treatment with fixed appliances.

## Abstract (pt)

**Objetivo:**

avaliar as percepções de pais e cuidadores com relação à qualidade de vida de adolescentes durante os primeiros 12 meses de tratamento ortodôntico com aparelhos fixos, e examinar as propriedades técnicas da versão brasileira do questionário *Parental-Caregiver Perceptions Questionnaire*(*P-CPQ*) no cenário ortodôntico.

**Métodos:**

dados de uma amostra de 96 pais e cuidadores de adolescentes em tratamento ortodôntico com aparelhos fixos foram coletados através do questionário *P-CPQ*. As avaliações foram realizadas antes da bandagem e colagem de braquetes (T_1_) e 12 meses após a colocação do aparelho fixo (T_2_). A análise estatística incluiu o teste Wilcoxon *signed rank *para o escore total do *P-CPQ* e a correção de Bonferroni para as subescalas do questionário. As propriedades técnicas do *P-CPQ* foram examinadas por meio do cálculo da responsividade e da mínima diferença clinicamente importante (MDCI).

**Resultados:**

dos 96 participantes, 76 eram mães de pacientes, 16 eram pais e 4 possuíam outra relação familiar. A idade média dos adolescentes foi de 11,49 ± 0,50 anos. A maioria das famílias tinha uma renda mensal igual ou menor a três salários mínimos brasileiros. Houve uma melhora significativa nas subescalas de bem-estar emocional e bem-estar social, que contribuíram para uma melhora do escore total de qualidade de vida (*p *< 0,001). As reduções dos escores do *P-CPQ* foram associadas com alterações clinicamente moderadas para o escore total e para as subescalas de bem-estar emocional e bem-estar social. A MDCI para o escore total do *P-CPQ* foi de 6,16.

**Conclusões:**

pais e cuidadores relataram uma melhora significativa na qualidade de vida de adolescentes em tratamento ortodôntico com aparelhos fixos.

## INTRODUCTION

The concept of oral health-related quality of life (OHRQoL) has been used to measure the impact of oral conditions on daily functioning and overall quality of life.^1^ In recent years, interest has focused on evaluating the OHRQoL of children and adolescents, since oral problems, such as dental caries and malocclusion, have an adverse impact on the physical and psychological well-being of young people.^2^^,^ 3

It has also been recognized that dental treatment has an impact on the OHRQoL of children and adolescents.^4^ Orthodontic treatment, for instance, can have a positive impact on quality of life after the appliance has been removed, as a result of improvements in one's emotional and social well-being. However, within the first months of therapy, the OHRQoL is negatively impacted due to worsening of oral symptoms and functional limitations.^5^ Thus, particular attention must be given to the first 12 months after bracket bonding,^6^ since disappointment and inconvenience on the part of patients and their parents/caregivers may lead to treatment dropouts.^7^^,^ 8

It is important to obtain information on parents'/caregivers' perception regarding orthodontic treatment of children and adolescents.^9^ Parents/caregivers play a major role in the success of ongoing treatment by encouraging compliance and cooperation during therapy and monitoring the hygiene and care required on the part of children/adolescents who wear fixed appliances.^10^ Moreover, parents'/caregivers' beliefs and values exert a major influence on treatment choices, since they are the main decision makers regarding the oral health of their sons/daughters.^11^ However, despite being relevant, information on parents'/caregivers' perception of the OHRQoL of adolescents during orthodontic treatment has been underinvestigated.^12^

The Parental-Caregiver Perceptions Questionnaire (P-CPQ) was developed to measure perceptions regarding the oral health of children and adolescents using parents/caregivers as proxies.^13^ The P-CPQ is a valid, reliable assessment tool that has been widely used in dental research;^13^^,^ 14 however, properties such as responsiveness and the minimal clinically important difference (MCID) have not been determined in studies involving parents/caregivers of adolescents undergoing orthodontic treatment with fixed appliances. Such properties should be evaluated in studies assessing the same population at different time periods throughout orthodontic treatment.^15^

The aim of the present study was to assess parents'/caregivers' perception of the OHRQoL of adolescents within the first 12 months of therapy with fixed appliances and investigate the evaluative properties of the P-CPQ in the orthodontic setting.

## METHODS

### Setting and sample

The sample comprised parents/caregivers of adolescents aged between 11 and 12 years old, scheduled for orthodontic treatment with fixed appliances at the Department of Pediatric Dentistry and Orthodontics of Universidade Federal de Minas Gerais (Brazil). Inclusion criteria were literacy and fluency in Brazilian Portuguese. Parents'/caregivers' schooling was collected through the standard Brazilian economic classification.^16^ To be included in this study, participants needed to have completed elementary education. Exclusion criteria were applicable to adolescents with: craniofacial anomaly, cognitive disorders, untreated dental caries, traumatic dental injury, poor gingival health and adolescents having undergone any dental treatment in the previous three months.

Sample size was determined using Sample Power 2.0 software (SPSS Inc., Chicago, IL, USA). Based on a pilot study, a sample of 85 participants would be required to identify a significant difference in OHRQoL between the first and second evaluations. Measures used for sample size calculation were a standard deviation of 19.50 at the first evaluation and 15.41 at the second evaluation. Power calculation was based on observed values in which the mean P-CPQ score changed by 10.34. Sample size was increased by 15 participants to compensate for potential losses (n = 100).

### Ethical issues

Ethical approval was obtained from the Institutional Review Board of the referred university under protocol #0421.0.203.000-11. Clarifications regarding the objectives and an assurance of confidentiality were given to the participants in the form of a written letter. Adolescents along with their parents/caregivers signed an informed consent form. All forms were numbered, but not identified with the participant's name. The key relating the codes to the names was stored in a locked cabinet to which only two researchers had access.

### Adolescents' malocclusion assessment

Adolescents' malocclusion was assessed by means of the Dental Aesthetic Index (DAI) which is a cross-cultural index consisting of ten components. Scores for each component were multiplied by a previously reported weight and a constant of 13 was added to obtain a total DAI score for each adolescent. Based on DAI scores, adolescents were classified into four categories of malocclusion with different orthodontic treatment needs assigned to each category: minor malocclusion/slight treatment need (DAI ≤ 25), definite malocclusion/elective treatment need (26 ≤ DAI ≤ 30), severe malocclusion/highly desirable treatment need (31 ≤ DAI ≤ 35), and handicapping malocclusion/mandatory treatment need (DAI ≥ 36).^17^

Oral examinations were performed by two trained and calibrated examiners. The calibration process consisted of a theoretical and a clinical step. The former involved a discussion on DAI, whereas the latter involved the examination of 15 adolescents who did not participate in the main study. To calculate intraexaminer agreement, adolescents were reexamined ten days later. Both steps were coordinated by an orthodontist with experience in epidemiological surveys. Kappa values for inter and intraexaminer agreement ranged between 0.84 and 0.90.

### OHRQoL assessment tool

OHRQoL was assessed by means of the P-CPQ which is a reliable, valid questionnaire that was developed in Canada.^13^ The P-CPQ has been cross-culturally adapted to be used on the Brazilian population, demonstrating adequate psychometric properties similar to those of the original instrument.^18^ This assessment tool consists of 31 items distributed among four subscales: oral symptoms (OS), functional limitations (FL), emotional well-being (EW) and social well-being (SW). Each item has five response options: "never" = 0; "once or twice" = 1; "sometimes" = 2; "often" = 3; and "every day or almost every day" = 4. A "don't know" response is also allowed. The overall P-CPQ score is computed by summing all four subscales item scores. Individual scores for each one of the four subscales can also be computed. The overall score ranges from 0 to 124, for which a higher score indicates a greater negative perception on the part of parents/caregivers regarding the OHRQoL of their adolescent sons/daughters.^13^

### Data collection

Data were collected by means of self-administered questionnaires in a 20-minute time frame. Parents/caregivers underwent two interviews. The first was held before banding and bonding of the fixed appliance for the determination of baseline data (T_1_). The second evaluation was held 12 months after the onset of orthodontic treatment (T_2_).

Treatment was conducted by postgraduate students in Orthodontics who stressed the positive effects and benefits of treatment to patients and their parents/caregivers. Shortly after the appliance was bonded, adolescents and their parents/caregivers were given a written description of the commitment required in terms of wearing the appliance, dietary restrictions and hygiene practices. This information was reemphasized in the subsequent appointments scheduled for appliance adjustments. The adolescents also received a supply of a standard non-medicated dental wax (Morelli^(r)^, Sorocaba, Brazil), were instructed to cover each bracket that led to mucosal irritation and reminded that, although painful and unpleasant, all ulcers would heal quickly. Parents/caregivers were encouraged to examine their personal schedules carefully before making appointments for their adolescent sons/daughters in order to maintain regular follow-up care. A phone number was provided in the event of an emergency if an attachment or a bracket came loose or a wire was broken.

### Statistical analysis

Statistical analysis was conducted by means of Statistical Package for the Social Sciences (SPSS for Windows, version 17.0, SPSS Inc., Chicago, IL, USA). Kolmogorov-Smirnov test revealed that P-CPQ scores exhibited non-normal distribution. Data analysis included descriptive statistics and Wilcoxon signed-rank test to determine the significance of differences in overall P-CPQ scores between T_1_ and T_2_. Significance level was set at 5% (*p* < 0.05). Additionally, Bonferroni correction was used to compare each one of the P-CPQ subscales between T_1_ and T_2_, with *p *values < 0.013 considered indicative of significance.

Responsiveness of P-CPQ was assessed by analyzing the effect size which is the difference between mean baseline (T_1_) and follow-up (T_2_) score divided by the standard deviation of the baseline score (T_1_). An effect size < 0.2 denotes a small clinically meaningful change, 0.2 to 0.7 indicates a moderate change and > 0.7 denotes a large change.^19^ To establish the MCID, the standard deviation of the outcome score at T_2_ was multiplied by 0.5.^20^ Having determined the MCID, the percentage of individuals presenting or exceeding this value was then computed.

## RESULTS

A total of 96 parents/caregivers of adolescents undergoing orthodontic treatment with fixed appliances participated in the present study (response rate: 96%). Four participants were excluded due to treatment dropouts or failure to fill out the follow-up questionnaire. Adolescents' mean age was 11.49 years (SD = 0.50). Most of the respondents were adolescents' mothers. Table 1 displays the sociodemographic characteristics of the sample and adolescents' orthodontic treatment needs. Table 2 presents the median and mode of the overall P-CPQ and subscales scores at T_1_ and T_2_. The median of the overall score, EW and SW scores were significantly lower at T_2_ in comparison to T_1_(*p* < 0.001). Table 3displays data on the mean overall and subscales scores at T_1_ and T_2_, the MCID, effect sizes and description of effect sizes. Reductions in scores were associated with effect size, demonstrating moderate clinically meaningful changes in the overall score as well as EW and SW subscale scores. The MCID was 6.16 for the overall P-CPQ score. Out of the 96 participants, 54 (56.3%) exceeded the MCID. Among those who exceeded the MCID, 41 (75.9%) reported improvement in perception regarding the overall OHRQoL of their adolescent son or daughter. 

**Table 1:** - Sociodemographic characteristics of sample and adolescents' orthodontic need.

	**Number (%)**
Respondents	
Mothers	76 (79.2)
Fathers	16 (16.7)
Other	4 (4.1)
Parents'/caregivers' schooling	
Elementary school	25 (26.0)
Middle school	16 (16.7)
High school	49 (51.0)
University degree	06 (6.3)
Family income (BMW/month)	
Up to 1 BMW	16 (16.7)
From 1 to 3 BMWs	54 (56.2)
From 3 to 5 BMWs	16 (16.7)
From 5 to 9 BMWs	08 (8.3)
More than 9 BMWs	02 (2.1)
Adolescents' sex	
Male	45 (46.9)
Female	51 (53.1)
Adolescents' age (years)	
11	49 (51.0)
12	47 (49.0)
Adolescents' orthodontic need	
Slight	35 (36.5)
Elective	26 (27.1)
Highly desirable	22 (22.9)
Mandatory	13 (13.5)

BMW = Brazilian Minimum Wage

**Table 2:** - Comparison of medians and modes of subscales scores and overall score at T_1_ and T_2_ among patients.

	**P-CPQ**	**Median**	**Mode**	**Median**	**Mode**	***p*-value **
	**range**	**T_1_**	**T_1_**	**T_2_**	**T_2_**	
OS	0 - 24	4	4	4	5	*p* = 0.087*
FL	0 - 32	5	2	4	0	*p* = 0.540*
EW	0 - 28	5	3	2	0	*p* < 0.001*
SW	0 - 40	4	0	2	0	*p* < 0.001*
OL	0 - 124	20	10	13	3	*p* < 0.001**

*Bonferroni correction. Significant at *p* < 0.013. **Wilcoxon test. Significant at *p* < 0.05. T_1_ = before fixed appliance placement; T_2_= 12 months after fixed appliance placement; OS = oral symptoms; FL = functional limitations; EW = emotional well-being; SW = social well-being; OL = overall score.

**Table 3:** - Mean and SD of subscale and overall scores at T_1_ and T_2_ with MCID and effect sizes.

	**Mean T_1_**	**SD T_1_**	**Mean T_2_**	**SD T_2_**	**MCID**	**Effect size**	**Effect size description**
OS	4.77	2.64	4.29	2.42	1.21	0.18	Small
FL	5.44	4.50	5.23	4.68	2.34	0.04	Small
EW	5.95	5.14	3.26	3.83	1.91	0.52	Moderate
SW	6.70	7.23	2.89	3.89	1.94	0.52	Moderate
OL	22.75	15.80	15.67	12.32	6.16	0.45	Moderate

SD = standard deviation. MCID = minimal clinically important difference. T_1_ = before fixed appliance placement; T_2_ = 12 months after fixed appliance placement; OS = oral symptoms; FL = functional limitations; EW = emotional well-being; SW = social well-being; OL = overall score.

## DISCUSSION

In the present study, parents/caregivers reported improvement in the overall OHRQoL of their adolescent sons/daughters at the end of the first twelve months of orthodontic treatment with fixed appliances. Although no statistical difference was found for OS and FL subscales, significant improvements were found in the EW and SW subscales. 

Orthodontic treatment is often associated with pain and discomfort caused by soft tissue irritation. Most mucosal lesions (erosion and ulceration) are related to trauma caused by the orthodontic appliance.^21^Individuals wearing fixed appliances may also experience limited oral functions. The most frequent complaints are impaired speech and chewing performance.^22^ In the present study, parents/caregivers reported no worsening of oral symptoms or functional limitations, but rather an enhanced sense of emotional and social well-being, which contributed to improve the perception of the overall OHRQoL of adolescents. There are two possible explanations for these findings. Firstly, improved emotional and social well-being may have occurred as a result of the positive perceptions regarding the fact that one's adolescent son/daughter has started treatment for malocclusions,^9^ of which presumed outcome is an improvement in dental esthetics,^23^ thereby providing social benefits for both adolescent patients and their parents/caregivers.^9^^,^ 24 Secondly, quality of life can be defined as the difference at a particular moment in time between the expectations and hopes of the individual and his/her current experiences.^25^ Parents have expectations regarding how their sons/daughters will be treated, the amount of pain to which they will be subjected and the effectiveness of treatment. Successful treatment is achieved by encouraging adolescents and their parents/caregivers to pursue an active, positive response to interventions by ignoring negative expectations or creating positive ones regarding treatment and healthcare services. Similarly, unmet expectations are likely to result in dissatisfaction.^26^ A poor outcome is more likely to occur when parents/caregivers do not encourage their son/daughter to attend appointments and adhere to the treatment regimen, such as the use of elastics.^27^ In the present study, the information provided by clinicians in relation to orthodontic treatment with fixed appliances may have provided a psychological rationale for the symptoms experienced during treatment, which were seen as temporary steps on the way to achieve the overall treatment goal. Thus, symptoms were reinterpreted as normative and, therefore, the divergence between expectations and experience was minimized.^26^

Both disease-specific and generic quality of life assessment tools must be reliable and valid. Ideally, they also need to be capable of identifying clinically important changes. Responsiveness is the ability of an assessment tool to detect changes in health status, whereas the MCID is used to interpret whether the observed change is important from the individual's or clinician's perspective.^28^ Based on effect size analysis, the responsiveness of the P-CPQ in detecting changes in parents'/caregivers' perception of the OHRQoL of adolescents submitted to orthodontic therapy with fixed appliances was adequate. The observed sensitivity to change was considered moderate for the overall score as well as the emotional and social well-being subscales. Researchers and clinicians should consider the P-CPQ as an adequate instrument for detection of changes over time and encourage its use.^15^ In the present study, the MCID demonstrated that a change of 6.16 points in the overall score is considered to be meaningful for parents/caregivers with regard to their level of satisfaction with orthodontic therapy of their adolescent sons/daughters. Moreover, this change should also be clinically detectable by orthodontists, so as to guide them during the course of treatment.^29^

The present study has limitations that should be recognized. Ideally, the psychosocial impact of orthodontic treatment with fixed appliances should be assessed by conducting a randomized clinical trial with a group of parents/caregivers of adolescents with malocclusion submitted to orthodontic treatment and a control group of parents/caregivers of adolescents with malocclusion receiving no treatment. However, this would not be feasible due to ethical concerns.^24^ Moreover, although some factors that could influence the outcome were controlled, such as the type of appliance worn; other factors were not controlled, such as differences regarding malocclusion severity, treatment complexity and the skill of clinicians who performed treatment.^30^

The results of the present study may be useful for clinical purposes. Quality of life measures have potential value in routine practice as means to prioritize problems, identify preferences, monitor changes and responses to treatment as well as facilitate communication between clinicians and both patients and their parents/caregivers.^31^ Such measures also allow clinicians to gain a better understanding of the magnitude of the benefits provided by orthodontic treatment with fixed appliances,^6^ and could also help orthodontists to discuss strategies with parents/caregivers of adolescents undergoing treatment.

## CONCLUSION

Parents/caregivers report improvements in the OHRQoL of their adolescent sons/daughters at the end of the first 12 months of therapy with fixed appliances. Parents/caregivers' opinion should be considered, as they may be aware of some variables that are key to orthodontic treatment outcomes.
